# Protein pyrophosphorylation by inositol pyrophosphates — detection, function, and regulation

**DOI:** 10.1002/1873-3468.70240

**Published:** 2025-12-02

**Authors:** Sarah Lampe, Tanmay Kumar Mohanty, Rashna Bhandari, Dorothea Fiedler

**Affiliations:** ^1^ Leibniz‐Forschungsinstitut für Molekulare Pharmakologie (FMP) Berlin Germany; ^2^ Institute of Chemistry Humboldt‐Universität zu Berlin Germany; ^3^ Laboratory of Cell Signalling, Centre for DNA Fingerprinting and Diagnostics Hyderabad India; ^4^ Graduate Studies Manipal Academy of Higher Education India

**Keywords:** back‐pyrophosphorylation, DDNL EThcD, enrichment, inositol pyrophosphates, P‐imidazolide, protein stability, pyrophosphoproteomics, pyrophosphorylation, rRNA synthesis, vesicle trafficking

## Abstract

Protein pyrophosphorylation is an emerging, unusual posttranslational modification. This signaling mechanism can be driven by inositol pyrophosphate messengers, which can convert a prephosphorylated protein to the corresponding pyrophosphoprotein. Endogenous protein pyrophosphorylation influences various cellular processes and signaling pathways, including the regulation of rRNA synthesis and the modulation of vesicular trafficking. Herein, we will summarize the current detection and analysis methods that have established the occurrence of pyrophosphorylation. These methods have also been used to explore the effects of pyrophosphorylation on protein structure and function. Putative mechanisms for the regulation of this intriguing, understudied modification will be discussed. Finally, the future needs for this developing area of signal transduction research are highlighted.

## Graphical abstract

Protein pyrophosphorylation is an unusual signaling mechanism that was discovered two decades ago. It can be driven by inositol pyrophosphate messengers and influences various cellular processes. Herein, we summarize the research progress and challenges of this field, covering pathways found to be regulated by this posttranslational modification as well as the development of analytical tools to detect pyrophosphorylation.

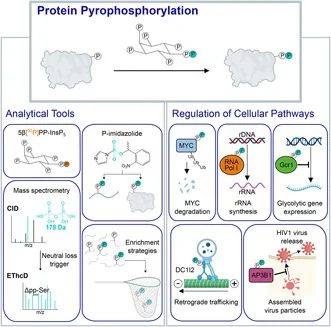

## Abbreviations

**1,5(PP)**_**2**_ **‐InsP**_**4**_, Bis‐1,5‐diphosphoinositol tetrakisphosphate

**1PP‐InsP**_**5**_, 1‐diphosphoinositol pentakisphosphate

**5PP‐InsP**_**5**_, 5‐diphosphoinositol pentakisphosphate

**5β[** ^**32**^ **P]PP‐InsP**_**5**_, 5‐diphosphoinositol pentakisphosphate labeled with ^32^P at the 5‐beta position

**ADP**, adenosine diphosphate

**AMP**, adenosine monophosphate

**AP3B1**, AP‐3 complex subunit beta‐1

**ATP**, adenosine triphosphate

**CID**, collision‐induced dissociation

**CK2**, casein kinase 2

**DC1I2**, dynein intermediate chain

**DCTN1**, dynactin subunit 1

**DDNL EThcD**, data‐dependent neutral‐loss‐triggered EThcD

**DIPP**, diphosphoinositol polyphosphate phosphohydrolase

**EThcD**, electron transfer/higher‐energy collision dissociation

**FBXW7**, F‐box/WD repeat‐containing protein 7

**FC**, fibrillar center

**GAPDH**, glyceraldehyde‐3‐phosphate‐dehydrogenase

**Gcr1**, glycolytic genes transcriptional activator Gcr1

**Gcr2**, glycolytic genes transcriptional activator Gcr2

**GFP**, green fluorescent protein

**GO**, gene ontology

**GTP**, guanosine triphosphate

**HCT116**, human colon tumor 116

**HIV**, human immunodeficiency virus

**IDR**, intrinsically disordered region

**InsP**, inositol polyphosphate

**InsP**_**6**_, inositol hexakisphosphate

**IP6K**, inositol hexakisphosphate kinase

**IRF3**, interferon regulatory factor 3

**Kcs1**, inositol hexakisphosphate kinase (yeast)

**KIF3A**, kinesin‐like protein KIF3A

**LPP**, lambda protein phosphatase

**MEF**, mouse embryonic fibroblast

**mRNA**, messenger ribonucleic acid

**MS**, mass spectrometry

**MYC**, Myc proto‐oncogene protein

**NAGK**, *N*‐acetyl‐d‐glucosamine kinase

**NM**, nuclear membrane

**NME1**, nucleoside diphosphate kinase A

**NOLC1**, nucleolar and coiled‐body phosphoprotein 1

**Nsr1**, nuclear localization sequence‐binding protein

**NUDIX**, nucleoside diphosphate linked to X

**PEST domain**, proline, glutamic acid, serine, threonine domain

**PGK1**, phosphoglycerate kinase

**P‐imidazolide**, phosphorimidazolide

**PIP**, phosphatidylinositol phosphate

**PM**, plasma membrane

**Pol I**, polymerase I

**PP1**, protein phosphatase 1

**PP2B**, protein phosphatase 2B

**PP2C**, protein phosphatase 2C

**PP‐InsP**, inositol pyrophosphate

**PPIP5K**, diphosphoinositol pentakisphosphate kinase

**PRM**, parallel reaction monitoring

**PTM**, posttranslational modification

**RAP1**, DNA‐binding protein rap1

**rDNA**, ribosomal deoxyribonucleic acid

**rRNA**, ribosomal ribonucleic acid

**SCAR**, single conservative amino acid replacements

**SILAC**, stable isotope labeling by amino acids in cell culture

**SIMAC**, sequential elution from immobilized metal affinity chromatography

**Srp40**, suppressor protein Srp40

**TCOF1**, treacle protein

**UAP1**, UDP‐*N*‐acetylglucosamine pyrophosphorylase 1

**UBF1**, upstream‐binding factor 1

**UDP‐GlcNAc**, UDP‐*N*‐acetylglucosamine

**UTP**, uridine triphosphate

**WT**, wild‐type

**YGR130c**, yeast protein (chromosome G, right arm, 130th open reading frame, coding strand)

Signaling *via* protein phosphorylation plays a central role in eukaryotic cell physiology, contributing to the regulation of a wide range of cellular processes [1, 2]. Besides the intensively studied phosphorylation events on serine, threonine, and tyrosine residues, the scope of these posttranslational modifications (PTMs) has been expanded to include phosphorylation on a variety of amino acid side chains (histidine, arginine, cysteine, aspartate, glutamate, lysine), highlighting the widespread use of this signaling mechanism and the need for sensitive and specific detection methods [3, 4, 5, 6, 7].

**Fig. 1:**
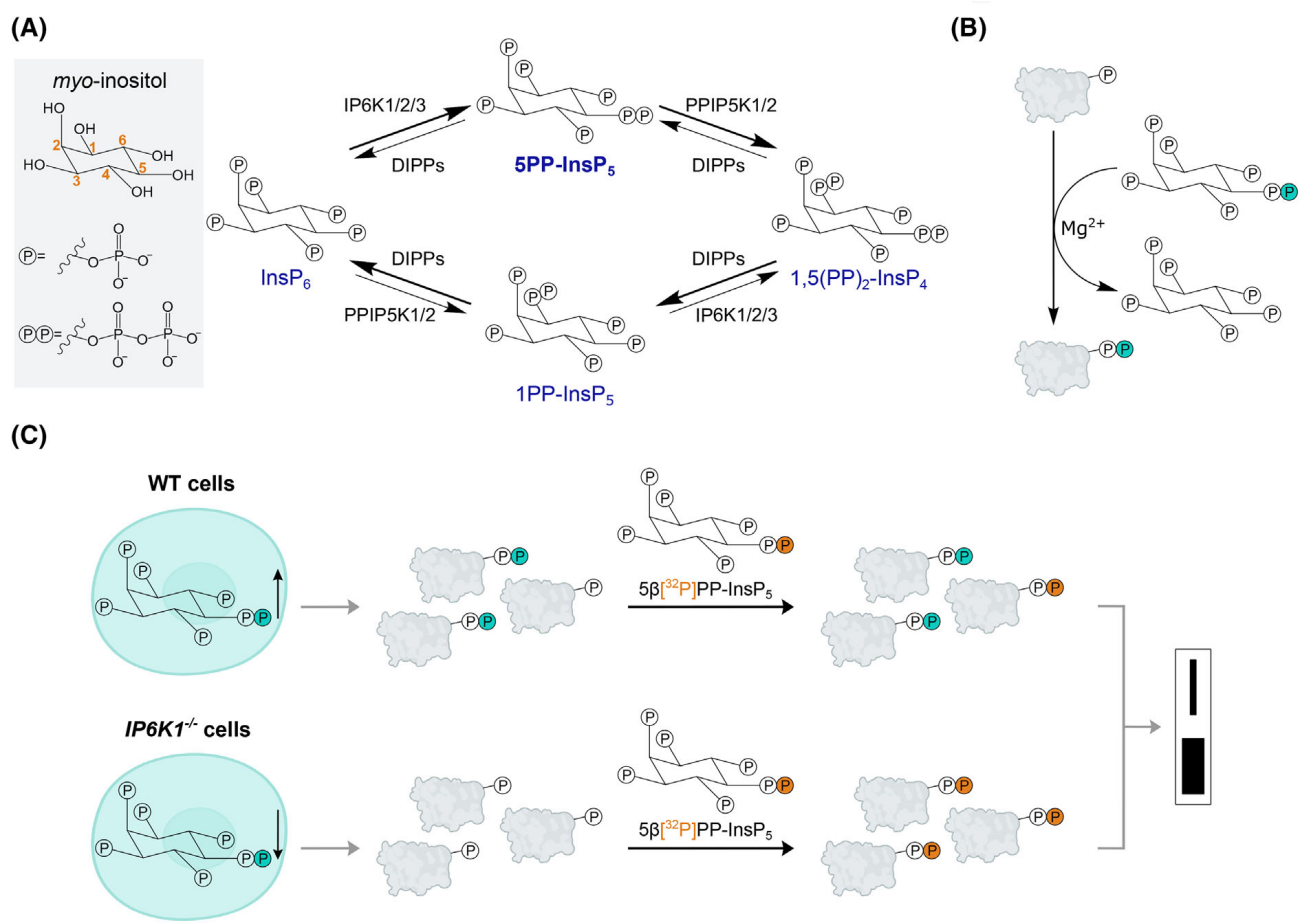
Protein pyrophosphorylation can be mediated by inositol pyrophosphates. (A) Biosynthetic pathway of inositol pyrophosphates in mammals starting from inositol hexakisphosphate (InsP_6_), which is converted to 5‐diphosphoinositol pentakisphosphate (5PP‐InsP_5_), 1‐diphosphoinositol pentakisphosphate (1PP‐InsP_5_), and bis‐1,5‐diphosphoinositol tetrakisphosphate (1,5(PP)_2_‐InsP_4_). The involved enzymes are inositol hexakisphosphate kinases 1/2/3 (IP6K1/2/3), diphosphoinositol polyphosphate phosphohydrolases 1/2α/2β/3α/3β (DIPPs), and the bifunctional enzymes diphosphoinositol pentakisphosphate kinase 1 and 2 that possess a kinase and a phosphatase domain to install and remove a β‐phosphoryl group (PPIP5K1/2). (B) Schematic illustration of pyrophosphorylation by 5PP‐InsP_5_ onto a prephosphorylated protein in the presence of magnesium (Mg^2+^). (C) Back‐pyrophosphorylation assay to detect *in vivo* PP‐InsP‐mediated pyrophosphorylation of target proteins. Some elements in the image have been obtained from BioRender, Lampe, S. (2025) https://BioRender.com/x0nwv6i.

The complexity of phosphoregulation was further increased by the discovery of protein pyrophosphorylation, an unusual PTM, in which a prephosphorylated serine or threonine residue is further phosphorylated to yield a pyrophosphoserine or pyrophosphothreonine group [8, 9, 10, 11]. The modification can be mediated by high‐energy inositol pyrophosphate (PP‐InsP) messengers, a group of highly charged signaling molecules that regulate key cellular processes, such as phosphate and energy homeostasis [11, 12, 13, 14, 15, 16, 17, 18, 19]. Biosynthetically, PP‐InsPs predominantly derive from inositol hexakisphosphate (InsP_6_, also known as phytate) downstream of the phosphatidylinositol phosphate (PIP) and inositol polyphosphate (InsP) metabolic pathway. The major enzyme groups responsible for the generation and rapid turnover of PP‐InsPs in mammals are inositol hexakisphosphate kinases (IP6Ks), diphosphoinositol pentakisphosphate kinases (PPIP5Ks), and diphosphoinositol polyphosphate phosphohydrolases (DIPPs). IP6Ks install a β‐phosphoryl group at the 5‐position, while PPIP5Ks are bifunctional enzymes that can install or remove the β‐phosphoryl group at the 1‐position of the *myo*‐inositol scaffold. In addition to the phosphatase domain of the PPIP5Ks, the phosphoanhydride bond of PP‐InsPs is selectively cleaved by DIPPs, which are part of the NUDIX hydrolase family (Fig. 1A) [20, 21, 22, 23, 24, 25].

## Discovery of protein pyrophosphorylation

The discovery of protein pyrophosphorylation goes back to a study published by Saiardi *et al*. in 2004 [8], aiming at deciphering the mechanism of action of PP‐InsP messengers, in particular of 5‐diphosphoinositol pentakisphosphate (5PP‐InsP_5_). When inositol pyrophosphates were initially characterized in 1993, researchers immediately noted the presence of the high‐energy phosphoanhydride bonds in the diphosphate moieties and proposed that inositol pyrophosphates may serve as phosphorylating agents [23, 26, 27].

To investigate whether 5PP‐InsP_5_ can, in fact, act as a phosphorylating agent, the Snyder laboratory prepared radiolabeled 5β[^32^P]PP‐InsP_5_, a molecule in which the β‐phosphoryl group is labeled with radioactive ^32^P, so that the putative transfer of this group could be monitored. The respective transfer was indeed observed when incubating 5β[^32^P]PP‐InsP_5_ with eukaryotic cell extracts from *Saccharomyces cerevisiae*, mice, and *Drosophila melanogaster*. Prominently radiolabeled proteins from *S. cerevisiae* were identified as Nsr1 (nuclear localization sequence‐binding protein), Srp40 (suppressor protein SRP40), and YGR130c. When these proteins were each overexpressed in *S. cerevisiae*, the phosphotransfer by 5β[^32^P]PP‐InsP_5_ was readily observed, underlining the proposal that 5PP‐InsP_5_ can donate phosphoryl groups onto serine side chains [8]. NOLC1 (nucleolar and coiled‐body phosphoprotein 1), the mammalian ortholog of Srp40, along with the related protein TCOF1 (treacle protein), was shown to undergo 5PP‐InsP_5_‐dependent phosphorylation as well. Interestingly, phosphotransfer by 5PP‐InsP_5_ appeared to selectively occur in eukaryotic cell extracts, as no proteins were radiolabeled in bacterial extracts. 5PP‐InsP_5_‐dependent phosphotransfer required a divalent cation as a cofactor, with magnesium ions (Mg^2+^) being preferred. In sum, these initial findings showed a phosphorylation of eukaryotic proteins by 5PP‐InsP_5_, which can take place *in vitro* in the absence of an enzyme. Supporting a potentially nonenzymatic reaction mechanism, the extent of the modification was shown to increase with rising temperatures up to 75 °C, a temperature at which most enzymes are likely denatured [8].

In 2007, Bhandari *et al.* [9] presented follow‐up work on 5PP‐InsP_5_‐driven phosphorylation, in which they disclosed that the phosphotransfer observed in 2004 surprisingly does not reflect a phosphorylation of a serine or threonine side chain, but instead entails the phosphorylation of a prephosphorylated residue, leading to the formation of a diphosphate group. To investigate 5PP‐InsP_5_‐mediated phosphotransfer on previously identified target proteins more thoroughly, the authors expressed protein substrates in *Escherichia coli*. Unexpectedly, unlike the proteins purified from yeast in 2004, the expressed proteins did not undergo modification by 5PP‐InsP_5_ [9]. Considering the fact that the proteins expressed in yeast may have been prephosphorylated by eukaryotic protein kinases (while they are unlikely to be phosphorylated in bacteria), the phosphorylation event was hypothesized to reflect a different type of modification that necessitates prephosphorylation. Further experiments unveiled that incubation of bacterially expressed proteins with yeast extract and ATP recovered the 5PP‐InsP_5_‐dependent modification of these proteins, whereas boiled yeast extract lacking functional protein kinases did not cause recovery. These results led to the proposal that 5PP‐InsP_5_ can transfer its β‐phosphoryl group exclusively to an already prephosphorylated residue, yielding diphosphate groups—also termed pyrophosphate groups (Fig. 1B) [9].

To identify putative protein kinases that catalyze the required prephosphorylation, the consensus motifs of the modified protein sequences were evaluated, revealing serine‐rich stretches interspersed with aspartic acid and glutamic acid residues. Therefore, the acidophilic Ser/Thr protein kinase casein kinase 2 (CK2) became a likely candidate. Consistent with this proposal, Nsr1, which also exhibits a corresponding strong consensus motif, was robustly phosphorylated by CK2 [9]. In addition, it was observed that prephosphorylation of target proteins can occur to a similar extent with GTP and ATP as nucleotide substrates. In contrast to most protein kinases, CK2 can use GTP as a phosphoryl donor to a comparable extent as ATP [9, 28]. Experiments with NOLC1 model peptides proved that the phosphotransfer by 5PP‐InsP_5_ is solely accepted by phosphoserine residues, and not by methylated phosphoserines or adjacent unmodified serine residues within the same peptide sequence [9].

Lastly, the modification generated by phosphotransfer from 5PP‐InsP_5_ is characterized by resistance to dephosphorylation by common protein phosphatases, such as lambda protein phosphatase (LPP), protein phosphatase 1 (PP1), and human DIPP1. These data strongly suggest that the 5PP‐InsP_5_‐dependent modification is different from canonical monophosphorylation [8, 9, 29].

Taken together, the results from the 2007 follow‐up study [9] revealed the presence of a previously unknown PTM generated by 5PP‐InsP_5_ in eukaryotes, namely diphosphate residues, which the authors termed protein pyrophosphorylation (Fig. 1B). The two pioneering studies of 2004 [8] and 2007 [9] led to the identification of a small set of pyrophosphorylated proteins from *S. cerevisiae* and mammalian cells [8, 9].

### Development of the back‐pyrophosphorylation assay

Although the discovery of protein pyrophosphorylation constituted a significant advancement in uncovering inositol pyrophosphate‐dependent signaling, the results were controversially discussed. The evidence for a nonenzymatic reaction mechanism was criticized, since all experiments were conducted *in vitro*, and could therefore not exclude the possibility that a kinase may be involved in a cellular setting. The detection method for pyrophosphorylation was also critiqued because work had to be carried out in an *in vitro* setting, thus not reporting on endogenous pyrophosphorylation levels [30, 31]. Consequently, Saiardi *et al*. developed a back‐pyrophosphorylation assay to determine whether a protein undergoes 5PP‐InsP_5_‐mediated pyrophosphorylation in intact cells [32]. The method compares the extent of 5β[^32^P]PP‐InsP_5_‐mediated pyrophosphorylation of target proteins overexpressed and purified from cell lines exhibiting either normal or low endogenous 5PP‐InsP_5_ levels. A target protein expressed in *IP6K1* ^−/−^ cells that contain low 5PP‐InsP_5_ levels is hypopyrophosphorylated, compared to the same target protein in wild‐type (WT) cells. The immunoprecipitated target protein is subsequently subjected to 5β[^32^P]PP‐InsP_5_‐incubation, in which the hypopyrophosphorylated protein gets pyrophosphorylated to a higher degree *in vitro*. A visualization by autoradiography provides a comparative readout that gives indirect evidence of PP‐InsP‐mediated pyrophosphorylation of proteins *in vivo* (Fig. 1C) [33]. This method has been applied successfully in several studies addressing the function of pyrophosphorylation for individual target proteins and has headed the research progress for years [10, 18, 32, 33, 34, 35].

## Analytical tools to detect and explore protein pyrophosphorylation

The reliable and unambiguous detection of a PTM is crucial to study its occurrence and its biological function and impact. Since the preparation of radiolabeled 5β[^32^P]PP‐InsP_5_ is complex, and handling of radiolabeled material requires regulatory clearances that limit the accessibility of this method, recent approaches have focused on creating non‐radioactive alternatives with direct readouts.

### Synthesis of pyrophosphopeptide standards

**Fig. 2:**
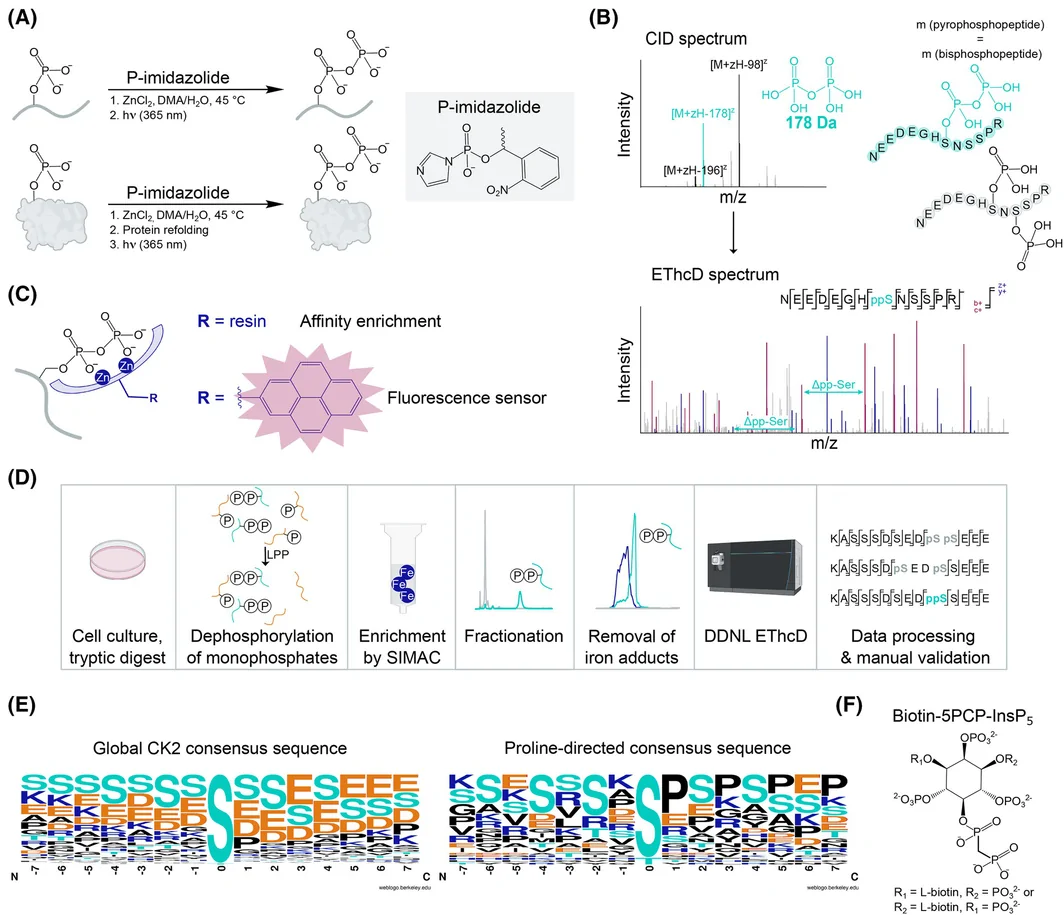
Tools to explore protein pyrophosphorylation. (A) Chemical pyrophosphorylation of peptides and proteins using photolabile phosphorimidazolide reagents, providing site‐specifically and stoichiometrically pyrophosphorylated standards. (B) Data‐dependent neutral‐loss‐triggered EThcD (DDNL EThcD) analysis for the unambiguous detection and identification of pyrophosphopeptides. Adapted from Morgan *et al*. (to view a copy of this license, visit http://creativecommons.org/licenses/by/4.0/) [10]. (C) Principle of a reagent for affinity enrichment or fluorescence detection of pyrophosphopeptides based on a dinuclear zinc(II) complex. (D) Pyrophosphoproteomic workflow, tailored to the enrichment and detection of tryptic pyrophosphopeptides followed by validation of pyrophosphosites. Adapted from Morgan *et al*. (to view a copy of this license, visit http://creativecommons.org/licenses/by/4.0/) [10]. (E) Consensus sequences of endogenous pyrophosphorylation sites derived from 148 pyrophosphosites of a pyrophosphoproteomic study, demonstrating a global CK2 consensus sequence (left), indicating CK2 as the common ‘priming’ kinase installing a monophosphate before pyrophosphorylation. Removal of CK2 consensus sites reveals a proline‐directed consensus sequence (right), which is associated with an alternative ATP‐dependent type of protein pyrophosphorylation. Consensus sequences are taken from Morgan *et al*. (to view a copy of this license, visit http://creativecommons.org/licenses/by/4.0/; sequence logos were generated using WebLogo) [10, 36]. (F) Immobilized and non‐hydrolysable PP‐InsP_5_ analogue for enrichment of interaction partners. Some elements in the image have been obtained from BioRender, Lampe, S. (2025) https://BioRender.com/x0nwv6i.

As an initial step toward various enrichment and detection strategies, a synthetic procedure to obtain pyrophosphorylated peptides was developed. The procedure relies on phosphorimidazolide (P‐imidazolide) reagents, which can be utilized to convert phosphopeptides into the corresponding pyrophosphopeptides. The reagents are compatible with a wide range of functional groups and do not require protection of other amino acid side chains. By utilizing a photolabile protecting group on the P‐imidazolide, the reagents are, in fact, compatible with whole proteins as substrates, given that the protein substrate can be properly refolded (Fig. 2A) [37, 38, 39]. Access to pyrophosphorylated peptides has subsequently enabled the development of enrichment and detection methods.

### Identification of pyrophosphorylation sites by mass spectrometry

With pyrophosphorylated standard peptides in hand and continuous advancements in mass spectrometry (MS) techniques, it was possible to evolve an MS analysis for the unambiguous identification of pyrophosphopeptides [40]. A huge analytical challenge was the distinction between pyrophosphorylated and bisphosphorylated peptides of the same sequence since they are isobaric, which means they have the same mass. For this reason, the method exploits that pyrophosphopeptides exhibit a characteristic neutral loss of −178 Da during collision‐induced dissociation (CID), which reflects the loss of pyrophosphoric acid and can therefore not occur for bisphosphorylated species. Whenever a neutral loss is detected, it triggers the selection of the same precursor for an electron transfer/higher‐energy collision dissociation (EThcD) fragmentation. The EThcD fragmentation results in extensive sequence coverage while preserving the labile modification (Fig. 2B) [40]. This approach allows the assignment of the exact position of pyrophosphorylation, which is particularly valuable given the occurrence of pyrophosphorylation in serine‐rich stretches [10, 40, 41].

### Enrichment of pyrophosphopeptides from complex samples

Because pyrophosphorylation involves two sequential phosphorylation events, the modification was expected to be sub‐stoichiometric. Furthermore, the high negative charge of the pyrophosphopeptides leads to poor ionization, and an enrichment prior to MS analysis appeared crucial. Therefore, an affinity reagent based on a dinuclear zinc(II) complex was developed. The complex retained diphosphate esters over other anionic groups, such as monophosphate esters and carboxylic acids, with high specificity. Immobilization of the complex provided a reagent capable of binding nanomolar quantities of pyrophosphopeptides. However, in the context of cell lysate samples, the immobilized zinc complex did not display the desirable capacity to be applied in proteomic experiments (Fig. 2C) [42, 43].

Alternatively, an enrichment using sequential elution from immobilized metal affinity chromatography (SIMAC) [44] was assessed and proved more successful in enriching pyrophosphopeptides from a complex sample, even though the high capacity is accompanied by low specificity, meaning that multiply phosphorylated peptides are enriched as well. Nevertheless, a multistep enrichment workflow was developed around a SIMAC. The workflow uses further strategies to reduce sample complexity and minimize competing mono‐ and multiply phosphorylated peptide species before the final LC–MS/MS measurement. For this purpose, a monophosphate‐specific phosphatase treatment with LPP and a fractionation are applied (Fig. 2D) [10, 44, 45]. Recently, the combination of the described data‐dependent neutral‐loss‐triggered EThcD (DDNL EThcD) analysis with the workflow tailored for the enrichment of tryptic pyrophosphopeptides from mammalian cell lysates led to the first direct identification of endogenous pyrophosphorylation sites on a proteome‐wide scale [10]. It should be noted that with this pyrophosphoproteomic strategy, despite LPP treatment, some isobaric bisphosphopeptides are also enriched, which underscores the necessity of an unambiguous assignment of pyrophosphorylation sites after MS measurement. Following this need a manual site validation is implemented after automated data processing to exclude false assignments caused by co‐elution and co‐fragmentation of isobaric bisphosphopeptides (Fig. 2D) [10].

### Application of pyrophosphoproteomics

The first pyrophosphoproteomic study [10] mentioned above enabled the unambiguous identification of 148 pyrophosphorylation sites across 71 human proteins. Intriguingly, the analysis highlighted NOLC1 and TCOF1, two of the initially identified pyrophosphoproteins [8, 9], as the most heavily pyrophosphorylated proteins known to date, containing 34 and 18 pyrophosphosites, respectively [10]. The alignment of all obtained pyrophosphorylation site sequences indicates and confirms CK2 as a central ‘priming’ kinase that installs the monophosphate residue prior to pyrophosphorylation by PP‐InsPs, as reflected by an acidophilic, serine‐rich consensus sequence (Fig. 2E). Additionally, the data reveal a high number of pyrophosphoproteins located in the nucleus and nucleolus [10, 46]. The pyrophosphoproteomic analysis opens up a broad view of protein pyrophosphorylation and will continue to annotate the human pyrophosphoproteome.

Also, the pyrophosphoproteomic strategy could be applied to any cell lysate, thereby holding the potential to reveal the pyrophosphoproteome of a variety of species. Apparent choices include lysates from *S. cerevisiae*, the organism in which protein pyrophosphorylation was first discovered [8, 9], as well as plant (e.g., *Arabidopsis thaliana*) and *Dictyostelium discoideum* lysates, in which PP‐InsPs are also involved in a variety of processes [47, 48, 49, 50, 51, 52, 53, 54, 55, 56]. Similarly, mammalian cell lines with perturbations in PP‐InsP biosynthesis can be investigated. Although 1,5‐bisdiphosphoinositol tetrakisphosphate (1,5(PP)_2_‐InsP_4_) can mediate pyrophosphorylation of proteins in an *S. cerevisiae* extract analogous to 5PP‐InsP_5_ *in vitro* [9], to examine the relevance of 1,5(PP)_2_‐InsP_4_ on endogenous pyrophosphorylation in mammalian cells, pyrophosphoproteomics was performed using lysates from *PPIP5K* ^−/−^ HCT116 cells [10]. Initial results revealed an equivalent number of pyrophosphosites compared to WT HCT116 cells, thereby indicating that 1,5(PP)_2_‐InsP_4_ does not play a crucial role in protein pyrophosphorylation *in vivo*. However, it must be taken into account that *PPIP5K* ^−/−^ cells not only lack 1,5(PP)_2_‐InsP_4_, but also exhibit increased levels of 5PP‐InsP_5_, which might compensate for any potential effect of the loss of 1,5(PP)_2_‐InsP_4_ on protein pyrophosphorylation [10, 57, 58, 59].

The pyrophosphoproteomic method has been exclusively executed using a tryptic digest, which excludes the detection of pyrophosphosites lying within very short or very large tryptic peptides. This is the case for the pyrophosphoprotein UBF1 (upstream‐binding factor 1) as its predicted pyrophosphosite(s) are located at its C‐terminus, which exhibits acidophilic CK2 consensus sequences, but only a few tryptic cleavage sites [10]. Further, the current method should be improved so that pyrophosphorylation levels can be quantified and stimulus‐specific changes in pyrophosphorylation can be monitored in the future.

Pyrophosphoproteomic analyses also shed light on a group of pyrophosphorylation sites that, unlike the majority, occur in structurally resolved regions and share a proline‐directed consensus sequence (Fig. 2E) [10]. To elucidate the effect of pyrophosphorylation on the structure and function of this group of proteins, access to site specifically and stoichiometrically pyrophosphorylated proteins was critical. Consequently, the kinases NAGK (*N*‐acetyl‐d‐glucosamine kinase) and NME1 (nucleoside diphosphate kinase A) were expressed using amber codon suppression to obtain the monophosphorylated species and subsequently converted to site‐specifically pyrophosphorylated proteins by conducting a chemoselective reaction with the P‐imidazolide reagent (Fig. 2A) [37, 38, 60, 61, 62]. Besides showing that phosphorylation and pyrophosphorylation alter their kinase activity and protein–protein interactions, it was observed that these phosphoproteins undergo ATP‐dependent autocatalytic phosphorylation reactions to generate the pyrophosphate modification [60, 62]. Thus, a second type of pyrophosphorylation mechanism was unveiled in addition to PP‐InsP‐mediated pyrophosphorylation. In principle, a similar biochemical strategy could be applied to investigate the function of ‘classical’, CK2‐guided pyrophosphorylation sites, but these modification sites are commonly located on large proteins within intrinsically disordered regions (IDRs), making their expression considerably more difficult. Furthermore, since ‘classical’ sites lie within serine‐rich regions, in which several pyrophosphorylation sites are frequently observed in close proximity, a multiply pyrophosphorylated protein would provide a better model for biochemical studies. Hence, a semi‐synthetic chemical ligation approach seems more promising to generate a suitably modified protein [63, 64, 65].

An alternative, indirect approach to identify potential target proteins of PP‐InsP‐mediated pyrophosphorylation is the enrichment of PP‐InsP binding partners using immobilized and non‐hydrolysable PP‐InsP analogues (Fig. 2F). Pulldown studies with *S. cerevisiae* lysates demonstrated that the presence of Mg^2+^ ions led to the enrichment of known pyrophosphorylation targets [66]. More recently, the affinity enrichment strategy was harnessed in mammalian cell lysates to identify additional pyrophosphorylation targets [67, 68].

There has been notable progress over the last two decades in dealing with the analytical challenges that protein pyrophosphorylation poses. The developed tools have enabled detection, identification, and enrichment of pyrophosphorylation sites, as well as the generation of standards at the peptide and protein levels. These methods now form the basis for the functional examination of this modification.

## Cellular pathways influenced by PP‐InsP‐dependent pyrophosphorylation

To date, there are only a handful of studies that have interrogated the impact of PP‐InsP‐dependent pyrophosphorylation on the function of the target protein, and by extension, the cellular pathways that are influenced by this modification. While these studies are described here, annotation of the pyrophosphoproteome is likely to bring more cellular functions under the umbrella of ‘pathways regulated by pyrophosphorylation’.

### Regulation of transcription

**Fig. 3:**
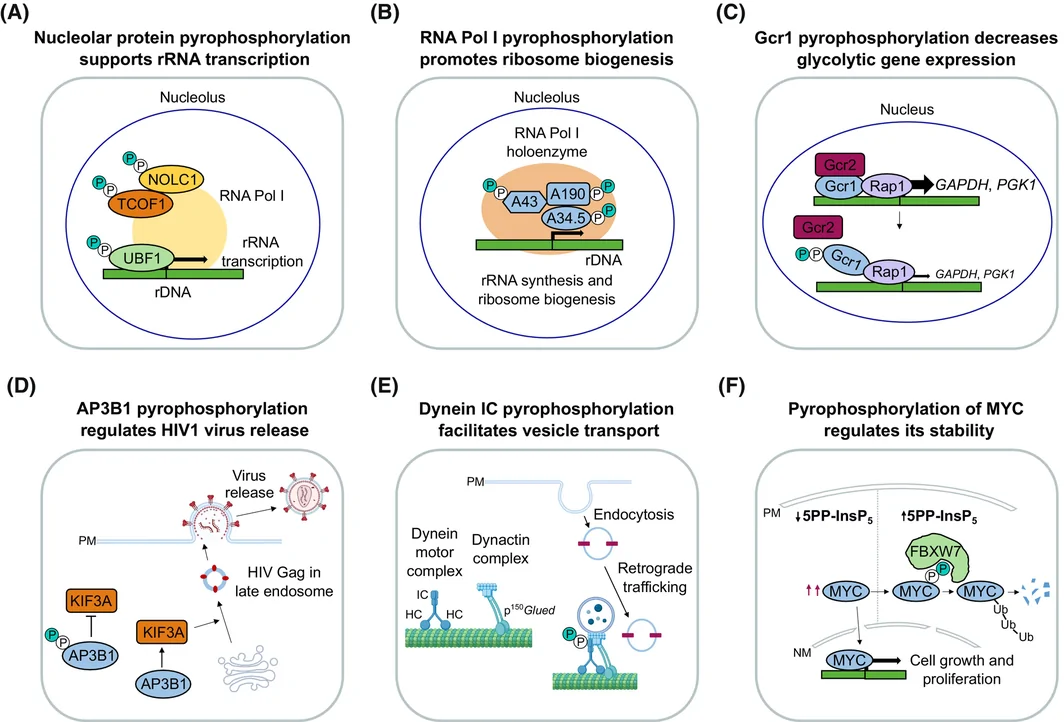
Pyrophosphorylation regulates different cellular pathways. 5PP‐InsP_5_ transfers its β‐phosphate to prephosphorylated proteins to modulate different cellular pathways. (A) Pyrophosphorylation of nucleolar proteins UBF1, NOLC1, and TCOF1 may modulate RNA polymerase I‐driven rRNA transcription in mammals. (B) Pyrophosphorylation of three *S. cerevisiae* RNA polymerase I subunits (A190, A43, and A34.5) promotes rRNA transcription elongation, which in turn regulates ribosome biogenesis. (C) In *S. cerevisiae*, Gcr1 transcription factor binds with its partners Rap1 and Gcr2 and upregulates the expression of glycolytic genes such as *GAPDH* and *PGK1*. Pyrophosphorylation of Gcr1 interrupts its binding with Gcr2, downregulating glycolytic gene expression. (D) HIV‐1 particle assembly requires endosomal trafficking of its structural protein Gag, which relies on the interaction between the host adaptor protein AP3 subunit AP3B1 and the kinesin motor protein KIF3A. Pyrophosphorylation of AP3B1 disrupts its interaction with KIF3A, downregulating the release of HIV‐1 virus‐like particles. (E) Pyrophosphorylation of the dynein intermediate chain IC (subunit of the dynein motor complex) promotes its interaction with p150^Glued^ (subunit of the dynactin complex) to facilitate dynein‐driven retrograde vesicle trafficking pathways. (F) The transcription factor MYC promotes cell growth and proliferation. Pyrophosphorylation of MYC facilitates its binding with the ubiquitin ligase FBXW7, leading to MYC polyubiquitination and its proteasomal degradation. All pyrophosphorylation‐based modes of regulation described here have been studied in mammals, except for (B) and (C), which occur in *S. cerevisiae*. Some elements in the image have been obtained from BioRender, Lampe, S. (2025) https://BioRender.com/x0nwv6i. PM, plasma membrane; and NM, nuclear membrane.

NOLC1 and TCOF1, the two human proteins with the highest number of pyrophosphorylation sites, are known to bind to each other and interact with RNA polymerase I (Pol I) in the nucleolus to regulate rRNA transcription [69, 70]. The pyrophosphoprotein UBF1, which is the transcription factor responsible for recruiting Pol I to the rDNA promoter, is localized exclusively to the nucleolar fibrillar center (FC), where Pol I‐dependent rRNA transcription takes place [71]. IP6K1 was shown to be enriched in this nucleolar subcompartment, prompting the hypothesis that 5PP‐InsP_5_ produced locally by IP6K1 must pyrophosphorylate these proteins in the nucleolus to influence rRNA synthesis [10]. This idea was tested using cells depleted for 5PP‐InsP_5_ by genomic deletion of IP6K1 and IP6K2, or by treatment with pan‐IP6K inhibitors. Indeed, in both cellular models, the levels of 45S pre‐rRNA showed an approximately two‐fold decrease, compared with controls [10] (Fig. 3A). These findings corroborated an earlier study, where *S. cerevisiae* lacking the IP6K homolog Kcs1 displayed reduced synthesis of 35S‐pre‐rRNA [46]. Three subunits of yeast Pol I (A190, A43, and A34.5) were found to undergo pyrophosphorylation by 5PP‐InsP_5_. Consequently, *kcs1*Δ yeast that lack 5PP‐InsP_5_ exhibit reduced Pol I elongation activity, lower ribosome levels, and impaired global protein synthesis (Fig. 3B). Another study, by the Saiardi laboratory, revealed that *kcs1*Δ yeast exhibit altered energy metabolism, including an increase in the ATP/ADP and ATP/AMP ratios [16]. They attributed this phenotype to 5PP‐InsP_5_‐mediated pyrophosphorylation of the yeast glycolytic transcription factor Gcr1. In *kcs1*Δ yeast, absence of Gcr1 pyrophosphorylation increases its interaction with Gcr2, enhancing the binding of the Gcr1‐Rap1 transcription factor complex to the promoters of glycolytic genes such as *GAPDH* and *PGK1*, leading to increased glycolysis in these cells (Fig. 3C). Together, these studies provided insights into the molecular mechanisms by which 5PP‐InsP_5_ functions as a metabolic regulator in eukaryotic cells [72, 73].

### Vesicle trafficking along microtubules

Another cellular function that is influenced by 5PP‐InsP_5_‐dependent pyrophosphorylation is the intracellular transport of vesicles along microtubule tracks. Cargo‐carrying vesicles moving from the cell center to the periphery toward the plus end of microtubules (anterograde trafficking) require the kinesin family of motor proteins, whereas vesicles moving from the cell periphery inwards to the minus end of microtubules (retrograde trafficking) depend on the motor protein dynein [74]. The Saiardi group demonstrated that pyrophosphorylation of AP3B1, the β‐subunit of the AP‐3 adaptor protein complex, disrupts its binding to the kinesin KIF3A [32]. To probe the functional consequences of AP3B1 pyrophosphorylation, they used *Ip6k1* ^−/−^ mouse embryonic fibroblasts (MEFs), which possess only 30% of the PP‐InsP_5_ levels present in *Ip6k1* ^+/+^ MEFs [9]. The AP‐3 complex binds Gag, a key structural protein in retroviruses like HIV‐1, and directs its intracellular trafficking and assembly into virus‐like particles that are released from the cell [75]. In *Ip6k1* ^−/−^ MEFs, diminished pyrophosphorylation of AP3B1 increases the binding of Gag with KIF3A, leading to enhanced release of HIV‐1 Gag virus‐like particles (Fig. 3D). In a separate study, *Ip6k1* ^−/−^ MEFs were shown to be defective for several dynein‐dependent trafficking pathways, including the movement of endocytosed vesicles, sorting of endocytosed cargo from early endosomes to recycling endosomes, and the maintenance of Golgi morphology [34]. All these phenotypes could be rescued by reintroducing catalytically active, but not inactive, IP6K1 into *Ip6k1* ^−/−^ MEFs, confirming that the synthesis of 5PP‐InsP_5_ by IP6K1 is necessary to maintain dynein‐dependent trafficking.

The slime mold *Dictyostelium discoideum*, which possesses 200 to 500‐fold more PP‐InsPs than mammalian cells [48], provided an excellent system to further examine the involvement of PP‐InsPs in dynein‐driven trafficking. Endosomes isolated from WT *D. discoideum*, or a strain lacking any detectable PP‐InsPs, were loaded onto polymerized microtubules, so vesicle movement could be monitored using an optical trap. The percentage of dynein‐driven microtubule minus‐end‐directed endosomes was significantly lower in the PP‐InsP‐depleted strain compared with WT, confirming that PP‐InsPs upregulate dynein‐dependent vesicle transport. Using the back‐pyrophosphorylation method combined with site‐directed mutagenesis, it was shown that 5PP‐InsP_5_ mediates the pyrophosphorylation of a single serine residue in an acidophilic cluster lying within the intrinsically disordered N‐terminal region of the dynein intermediate chain (DC1I2), a subunit of the dynein motor complex. DC1I2 is known to mediate the binding of dynein to its cofactor dynactin, which is crucial to maintain dynein processivity, activation, and cargo binding [76]. In *Ip6k1* ^−/−^ MEFs, reduced pyrophosphorylation of DC1I2 led to a decrease in its binding with the p150^Glued^ subunit of dynactin (DCTN1), providing an explanation for impaired dynein‐driven trafficking observed in these cells (Fig. 3E).

### Regulation of protein stability

The preference of PP‐InsPs for acidophilic serine‐rich sequences as targets for pyrophosphorylation was used to identify a pyrophosphorylated protein of clinical significance—the oncoprotein MYC [18]. This short‐lived transcription factor, upregulated in many cancers, is a master regulator of cell proliferation, differentiation, and apoptosis [77, 78]. MYC possesses a disordered PEST domain (enriched in Pro, Glu/Asp, Ser, and Thr residues), which promotes its rapid turnover. The back‐pyrophosphorylation method was used to confirm endogenous MYC pyrophosphorylation, and the region of pyrophosphorylation was mapped to one or more of a cluster of three Ser residues lying within the PEST domain of MYC. Replacing these three Ser residues with Thr allowed for phosphorylation of this sequence by the protein kinase CK2, but not its pyrophosphorylation by 5PP‐InsP_5_. This 3(S/T) mutant version of MYC was more stable compared with the native protein, due to its reduced polyubiquitination‐dependent degradation. Attaching the native PEST domain of MYC onto GFP reduced the stability of this normally very stable reporter, but the 3(S/T) mutant PEST domain had no impact on GFP stability. This GFP‐tagged MYC PEST domain was used as a handle to unearth the molecular basis for the regulation of MYC stability by pyrophosphorylation. When tagged to GFP, the native MYC PEST domain acted as a ‘pyrophosphodegron’, binding with the E3 ubiquitin ligase FBW7α (F‐box/WD repeat‐containing protein 7, FBXW7), and leading to the degradation of GFP. The 3(S/T) mutant MYC PEST domain did not display this ability. In *Ip6k1* ^−/−^ MEFs, reduced MYC pyrophosphorylation led to its decreased polyubiquitination and increased stability, and in lymphocytes derived from spleens of *Ip6k1* ^−/−^ mice, increased MYC levels correlated with a 50% enhancement in stimulus‐induced proliferation (Fig. 3F).

A recent study reported that UAP1 (UDP‐N‐acetylglucosamine pyrophosphorylase 1), the enzyme that drives the synthesis of UDP‐N‐acetylglucosamine (UDP‐GlcNAc) from UTP and GlcNAc‐1‐P, can catalyze the transfer of the β‐phosphoryl group from 5PP‐InsP_5_ to prephosphorylated IRF3 (interferon regulatory factor 3), a transcription factor involved in the innate immune response against viral infections [79]. Pyrophosphorylation on IRF3 was shown to promote its dimerization, nuclear translocation, and subsequent function as a transcription activator. It remains to be tested whether UAP1 can also catalyze 5PP‐InsP_5_‐mediated pyrophosphorylation on other proteins.

One common theme that emerges from the above descriptions of the molecular mechanisms by which protein pyrophosphorylation impacts different cellular functions is that this modification can regulate protein–protein interactions. Pyrophosphorylation of Gcr1 and AP3B1 disrupts their binding with Gcr2 and KIF3A, respectively, whereas pyrophosphorylation of DC1I2 and MYC increases their binding to DCTN1 and FBXW7, respectively [16, 18, 32, 34]. In this regard, pyrophosphorylation is similar to phosphorylation and other PTMs, which can modulate the interaction of the modified protein with its binding partners. With the annotation of the human pyrophosphoproteome, it is only a matter of time until we identify how this modification affects other aspects of protein function, such as activity, localization, or structure. The few examples of regulation of cell function by PP‐InsP‐dependent pyrophosphorylation described in this section are likely just the ‘tip of the iceberg’. Gene ontology (GO) analysis of the pyrophosphoproteome suggests that this modification may regulate several additional biological processes, including mRNA splicing, RNA transport, and chromatin organization [10], paving the way for future investigations on the connection between protein pyrophosphorylation and these cellular processes.

## Regulation of protein pyrophosphorylation

Ever since the discovery that the β‐phosphoryl group of a PP‐InsP can transfer to a protein, a major question has remained—how does the cell regulate this protein modification? It turns out that several conditions need to be met for this phosphoryl transfer to happen.

### Requirement of Mg^2+^ to drive pyrophosphorylation

One essential requirement for PP‐InsP‐mediated protein pyrophosphorylation is the presence of the divalent cation Mg^2+^. A CK2 prephosphorylated protein can undergo *in vitro* pyrophosphorylation in the presence of as low as 0.5–1 μm radiolabeled 5β[^32^P]PP‐InsP_5_ [10, 11, 18, 33]. These reactions appear to be most efficient in the presence of 6 mm Mg^2+^ ions. The reactions proceed with lower conversion when 1 mm Mg^2+^ ions are added, and no conversion is observed in the presence of 10 or 100 μm Mg^2+^ ions [11]. The reason for the several thousand‐fold molar excess of Mg^2+^ over PP‐InsP during protein pyrophosphorylation remains enigmatic. One likely mechanism by which Mg^2+^ ions work is by complexation of the phosphoryl groups of PP‐InsP on one side and the carboxyl side chains of Asp/Glu residues in the substrate protein on the other side, thereby bringing the two reactive partners into close proximity. In the cell, freely available Mg^2+^ ions, believed to be around 0.5–1 mm [80], may suffice for this bridging function. In this way, cells could modulate the availability of free Mg^2+^ to regulate PP‐InsP‐driven protein pyrophosphorylation.

### Conformation of PP‐InsPs

Coordination with Mg^2+^ has also been shown to influence the conformational switching of PP‐InsPs from the typical conformation, in which all positions, except the 2‐position, are orientated equatorially, to a ‘flipped’ conformation, in which five of the phosphate/diphosphate moieties are arranged axially, above and below the plane of the inositol ring [81, 82]. This conformational transition is also dependent on the pH of the milieu, where deprotonation of the phosphoryl groups on the inositol ring at higher pH favors a switch to the ‘flipped’ conformation in which the negative charges are distributed over a larger overall area. In this context, it has been shown that pyrophosphorylation of Nsr1 by 5β[^32^P]PP‐InsP_5_ is more efficient at pH 5 and 6 compared with pH 8 and 9 [11]. Further studies are needed to pinpoint which conformation of PP‐InsPs favors phosphotransfer to a prephosphorylated Ser/Thr residue, and what role Mg^2+^, the local pH, and the sequence of the target protein play in the process.

### Sequence composition of target proteins

The other major factor that influences the ability of a substrate protein to undergo PP‐InsP‐dependent pyrophosphorylation is its own sequence. Unlike protein‐kinase‐dependent phosphorylation sites that follow a linear consensus sequence pattern [83], sites for PP‐InsP‐mediated pyrophosphorylation appear to have a consensus composition (Fig. 2E). Of the 148 pyrophosphosites identified by pyrophosphoproteomics, 145 were on Ser residues, and only three pyrophosphorylated Thr residues were detected, of which one (on NME1) is already known to occur *via* a mechanism independent of PP‐InsPs [62]. In the case of MYC, as described above, substitution of Ser with Thr abrogated pyrophosphorylation. It is unclear why Ser is preferred over Thr as an acceptor of the β‐phosphoryl group from PP‐InsPs. A second aspect of the pyrophosphosites mapped by mass spectrometry is their predominant location within acidophilic serine stretches. From the 148 mapped sites, 128 were classified as potential substrates for acidophilic Ser/Thr kinases such as CK2 [10]. The potential importance of carboxyl side chains in the vicinity of the target Ser in the context of coordination with Mg^2+^ ions is discussed above. It is still not clear whether the pyrophosphosites that lie in regions that are not acidophilic, other than the sites on NAGK and NME1 that are known to be ATP‐dependent [60, 62], undergo pyrophosphorylation by PP‐InsPs or *via* another mechanism. A third important aspect of the sequences in which pyrophosphosites are located is their extent of structural disorder. From all pyrophosphosites identified by mass spectrometry, 91% are located within a continuous stretch of 20 or more residues classified as an intrinsically disordered region [10]. One possible reason for disorder in a pyrophosphorylation substrate is its accessibility and structural flexibility, which would allow the target sequence to adopt a conformation suited for coordination with Mg^2+^‐PP‐InsP, positioning the acceptor phospho‐Ser in close proximity to the β‐phosphoryl group of the donor PP‐InsP. This scenario envisages ‘self‐catalysis’ of pyrophosphorylation by the target protein in the absence of another enzyme.

### Interaction of IP6K with pyrophosphorylation substrate proteins

**Fig. 4:**
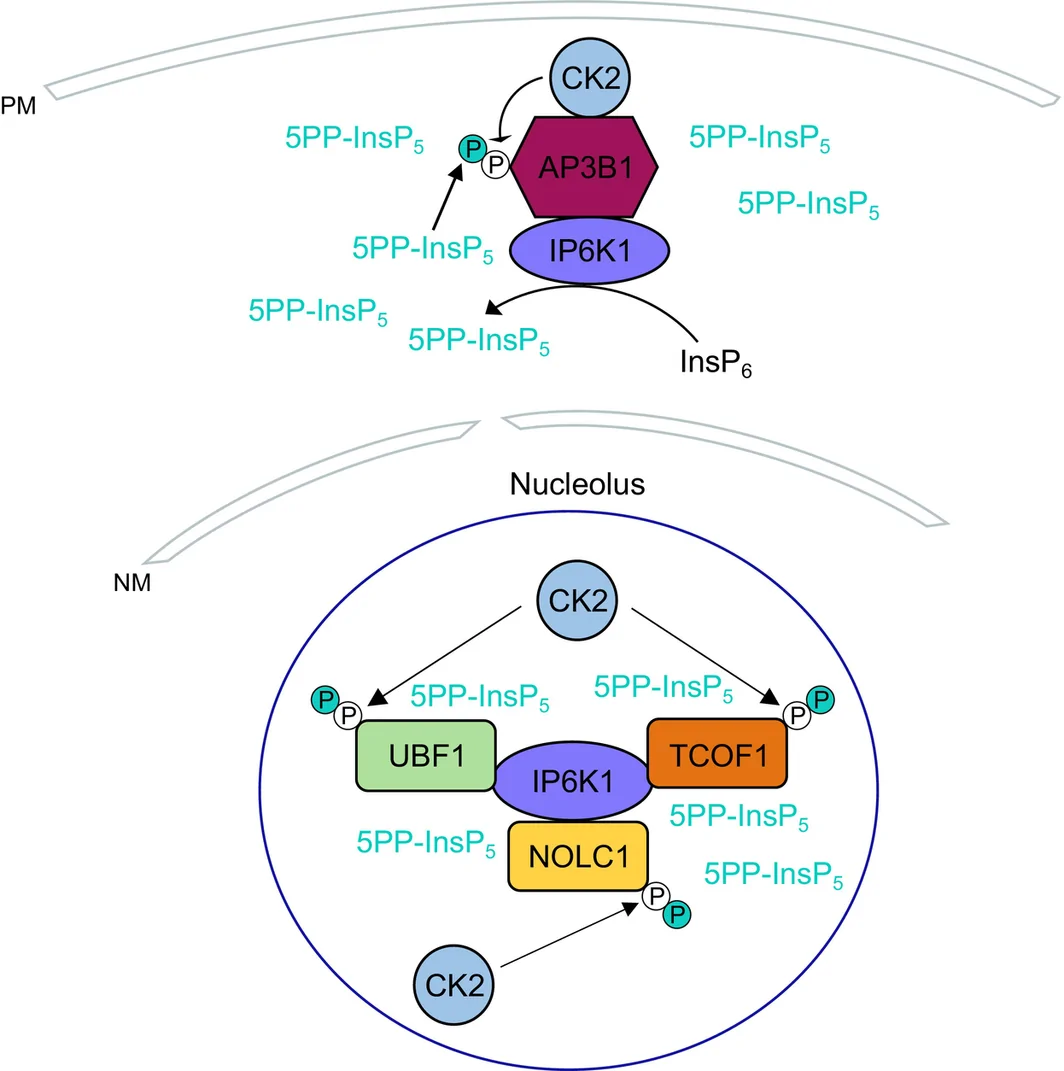
Regulation of 5PP‐InsP_5_‐mediated protein pyrophosphorylation. In the cytoplasm, the pyrophosphorylation substrate AP3B1 interacts with both CK2 and IP6K1. Interaction of AP3B1 with CK2 facilitates its phosphorylation, which is essential for subsequent pyrophosphorylation. IP6K1 interaction with AP3B1 increases local 5PP‐InsP5 synthesis to facilitate AP3B1 pyrophosphorylation. Likewise, in the nucleolus, CK2 pre‐phosphorylates UBF1, NOLC1, and TCOF1. Subsequent 5PP‐InsP5–mediated pyrophosphorylation of these proteins is likely to be facilitated by their interaction with IP6K1. NM, nuclear membrane; PM, plasma membrane.

Finally, if PP‐InsP‐dependent protein pyrophosphorylation is driven by mass action, it must require a high local concentration of the donor PP‐InsP in the vicinity of the acceptor substrate protein. For pyrophosphorylation to happen, the local concentration of the donor PP‐InsP must also exceed that of InsP_6_, which has a 5 to 50‐fold higher overall intracellular concentration compared with PP‐InsPs [84], and has been shown to inhibit 5PP‐InsP_5_‐mediated pyrophosphorylation *in vitro* [8]. A recent study demonstrated how such a high local concentration of PP‐InsP may be generated. The protein interactome of IP6K1 was shown to include known pyrophosphorylated proteins, including NOLC1, TCOF1, UBF1, and AP3B1, and the prephosphorylating kinase CK2 [35]. IP6K1 was found to interact directly with AP3B1, and indirectly with the catalytic α‐subunit of CK2, which in turn directly binds and phosphorylates AP3B1. A disordered region lying within the N‐lobe of IP6K1 is the predominant motif responsible for the binding of IP6K1 to AP3B1. Overexpression of this fragment of IP6K1 had a dominant negative effect on the binding between IP6K1 and AP3B1, and this phenomenon, along with the back‐pyrophosphorylation method, was employed to demonstrate that the interaction between IP6K1 and AP3B1 promotes AP3B1 pyrophosphorylation inside cells (Fig. 4). All three nucleolar pyrophosphorylation substrates associated with IP6K1—UBF1, TCOF1, and NOLC1—are known to be associated with, and phosphorylated by, CK2 [85, 86, 87, 88, 89]. CK2 has also been shown to phosphorylate these proteins *in vitro* prior to their pyrophosphorylation by 5PP‐InsP_5_ [10] (Fig. 4). In this way, cells may up‐ or down‐regulate the formation of a tripartite complex between the pyrophosphorylation substrate, a priming kinase like CK2, and IP6K1 (or other IP6Ks), to increase or decrease pyrophosphorylation of the target protein. Regulation of the enzyme activity, levels, and localization of CK2 and IP6K would also provide different ways in which to control the extent of prephosphorylation and pyrophosphorylation of a target protein.

## Conclusion and outlook

Protein pyrophosphorylation was discovered almost 20 years ago, yet we are just beginning to unravel the scope and physiological impact of this unusual modification. While the initial detection strategies of protein pyrophosphorylation using radiolabeled 5β[^32^P]PP‐InsP_5_ have provided fundamental insights into the function and regulation of this PTM on individual target proteins [10, 18, 32, 33, 34, 35], they also have limitations. Besides its limited accessibility and short shelf‐life, radiolabeling with 5β[^32^P]PP‐InsP_5_ is unable to determine the specific site of modification. The continuous development of analytical tools was necessary for the detection and localization of endogenous modification sites.

A synthetic route toward stoichiometrically pyrophosphorylated peptides and proteins has been instrumental in investigating the chemical and biochemical properties of this modification [37, 38, 39]. Furthermore, access to pyrophosphopeptide standards was key for the development of an affinity reagent [42, 43] as well as a mass spectrometry method [10, 40, 41]. The latter allowed for the unambiguous detection of pyrophosphopeptides and their specific pyrophosphorylation site, reflecting a major advancement [10, 40, 41]. Since the affinity reagent, based on a dinuclear zinc(II) complex [42, 43], was insufficient for the enrichment of pyrophosphopeptides from complex samples, an extensive enrichment workflow was developed, which complements the MS method for pyrophosphoproteomic analyses [10]. The application of the pyrophosphoproteomic strategy to two human cell lines recently uncovered almost 150 endogenous pyrophosphosites, predominantly on nuclear and nucleolar proteins [10]. Since the method is fairly extensive and requires large amounts of lysate, the enrichment and MS workflow for pyrophosphorylated peptides should be further optimized to expand our understanding of protein pyrophosphorylation and its occurrence. For example, enrichment using nuclear or nucleolar isolates could reduce sample amount, complexity, and competition with non‐pyrophosphorylated cytosolic peptides. In addition, the implementation of alternative digestive enzymes should be considered to access pyrophosphosites in regions lacking tryptic cleavage sites.

As for now, pyrophosphoproteomics was exclusively applied to human cell lysates [10]. Other species, such as *Dictyostelium discoideum* and plants that possess a conserved and intensively studied PP‐InsP metabolism [47, 48, 49, 50, 51, 52, 53, 54, 55, 56], appear predestined to also exhibit PP‐InsP‐driven protein pyrophosphorylation. Thus, their putative pyrophosphoproteomes represent a research gap that remains to be filled. Although the loss of 1,5(PP)_2_‐InsP_4_ in HCT116 cells seemed not to appreciably alter endogenous protein pyrophosphorylation [10], the importance of this PP‐InsP, which is especially abundant in *Dictyostelium discoideum* [48], in driving protein pyrophosphorylation should be explored.

To establish protein pyrophosphorylation as a dynamic signaling event, quantitative detection methods will be highly desirable. An initial quantification of a pyrophosphorylation site was reported very recently, using a SCAR (single conservative amino acid replacement) standard peptide. When spiked into cell lysate, a notable amount of pyrophosphorylation on NME1 could be quantified using PRM (parallel reaction monitoring) [37, 39, 62, 90]. We envision that PRM can be applied more globally to target a variety of known pyrophosphopeptides as well. When this is applied in combination with SILAC (stable isotope labeling by amino acids in cell culture), this would allow for annotation of stimulus‐specific changes of pyrophosphorylation levels [91, 92].

In addition to quantifying pyrophosphorylation levels, the scope of detectable sites should also be expanded. The current MS method is not able to detect pyrophosphorylation on residues other than serine and threonine residues (i.e., tyrosine). Also, current approaches focus on the detection of pyrophosphopeptides when monophosphorylation has been depleted [10]. To elucidate potential cross‐regulation between phosphorylation and pyrophosphorylation, an additional set of synthetic standard peptides will be necessary to characterize the corresponding enrichment, chromatography, and MS properties. To address the high level of complexity that is encoded by phosphorylation and pyrophosphorylation at the protein level, site‐specifically and stoichiometrically modified (pyro)phosphorylated proteins should be generated. While single pyrophosphorylation has already been successfully introduced [60, 62], the incorporation of multiple modification sites likely necessitates the application of alternative methods, such as expressed protein ligation [93, 94].

NOLC1 and TCOF1 currently appear to be the two most heavily pyrophosphorylated mammalian proteins. These proteins were among the handful of initially identified proteins that were modified using radiolabeled 5β[^32^P]PP‐InsP_5_, underscoring the utility of the radiolabeling method [8, 9, 10]. To extend the accessibility of the *in vitro* labeling studies with PP‐InsPs, one could imagine a non‐radioactive analog, for example, an inositol pyrophosphate carrying a β‐thiophosphate or an ^18^O‐labeled β‐phosphate as recently reported by Jessen *et al*. [95, 96]. For user‐friendly, non‐radioactive detection of pyrophosphorylation, a pan‐specific pyrophosphoserine antibody would be of great use, opening up new possibilities for functional and mechanistic studies. Such an antibody, if available commercially, would empower every cellular biochemist to study their favorite protein, pathway, or model organism for regulation by pyrophosphorylation, potentially increasing the list of biological functions influenced by this unique modification. For example, one class of organisms in which the actions of PP‐InsPs have been studied extensively, but nothing is known yet about their ability to pyrophosphorylate proteins, is the plant kingdom [47]. Pyrophosphorylation by PP‐InsPs may rely on a variety of catalytic mechanisms depending on the target site, which may include enzyme‐dependent and enzyme‐independent reactions. Future studies to understand the reaction mechanism and kinetics of pyrophosphorylation could rely on biophysical methods to stoichiometrically and structurally track this phosphoryl transfer using singly prephosphorylated peptide substrates and ^18^O‐labeled β‐phosphate containing PP‐InsP donors.

PP‐InsP‐dependent pyrophosphorylation always occurs in the context of protein kinase‐dependent phosphorylation at the same residue. While the challenge of detecting pyrophosphorylation within heavily phosphorylated regions of a protein by mass spectrometry has been tackled, the impact of phosphorylation *vs* pyrophosphorylation on the functions of the target protein, and its consequence in the cellular context, remains to be systematically addressed. In the case of MYC, pyrophosphorylation, along with phosphorylation in the PEST domain, was shown to facilitate interaction with the E3 ubiquitin ligase FBW7α, and augment MYC polyubiquitination and degradation [18]. Similar studies on other individual substrate proteins will reveal how pyrophosphorylation fits into the complex pattern of PTMs that regulate protein function.

Drawing parallels between protein phosphorylation and pyrophosphorylation also brings up the question of enzymes that catalyze these modifications. While PP‐InsP‐dependent pyrophosphorylation can occur *in vitro* without an enzyme, the possibility of UAP1 or other enzymes facilitating this modification *in vivo* remains to be thoroughly investigated. A question that is often asked about protein pyrophosphorylation is ‘how does it come off the protein?’. Early studies on *in vitro* pyrophosphorylated Nsr1 suggested that pyrophosphorylation is resistant to a few tested mammalian Ser/Thr phosphatases, including PP1 and PP2B (protein phosphatase 2B) [9]. Indeed, one mechanism by which pyrophosphorylation of a protein at a specific site may modulate its function differently than phosphorylation at the same site is by rendering that site inert to dephosphorylation by its canonical phosphatase. In a subsequent quantitative study using synthetic pyrophosphopeptides corresponding to pyrophosphorylated sequences in AP3B1, NOLC1, Nsr1, and Gcr1, these were confirmed to be resistant to PP1 and PP2C (protein phosphatase 2C) [29]. These peptides were, however, sensitive to alkaline phosphatases, and the peptides were dephosphorylated in the presence of yeast or mammalian cell extracts, but not by heat‐denatured lysates [29]. These experiments with peptide substrates hint at the presence of cellular (pyro)phosphatases waiting to be uncovered. Identification and characterization of such (pyro)phosphatases will be an important step in understanding the regulation of protein pyrophosphorylation. These (pyro)phosphatases will be important tools to investigate endogenous protein pyrophosphorylation, as pyrophosphorylation levels could be controlled directly by genetically perturbing said phosphatases.

Mass spectrometry evidence of protein pyrophosphorylation has established this PTM as one of the bona fide mechanisms by which PP‐InsPs regulate cell function. Recent discoveries of ATP‐dependent and PP‐InsP‐independent pyrophosphorylation have opened new areas of study centered around this unique modification. In the coming years, we hope that advancements in detection methods will bring the study of protein pyrophosphorylation to every lab bench, so that examining pyrophosphorylation will become routine practice for investigators studying PTMs on their proteins of interest.

## Author contributions

Conceptualization, RB and DF; writing – original draft, SL, TKM, RB and DF; writing – review and editing, SL, TKM, RB and DF; visualization, SL and TKM; supervision, RB and DF; funding acquisition, RB and DF. All authors have read and agreed to the published version of the manuscript.
